# Clinical and biochemical changes in 53 Swedish dogs bitten by the European adder - *Vipera berus*

**DOI:** 10.1186/1751-0147-52-26

**Published:** 2010-04-23

**Authors:** Jessica Berger Lervik, Inger Lilliehöök, Jan HM Frendin

**Affiliations:** 1 Södra Animal Hospital, Månskärsvägen 13, S-141 75 Kungens Kurva, Sweden; 2 Laboratory of Clinical Pathology, University Animal Hospital, Swedish, University of Agricultural Sciences, PO box 7070, S-750 07 Uppsala, Sweden; 3 Dept of Clinical Sciences, Section of Anaesthesiology and Emergency and Critical Care, Swedish University of Agricultural Sciences, PO box 7070, S-750 07 Uppsala, Sweden

## Abstract

**Background:**

Every year many dogs in Sweden are bitten by *Vipera berus*, the only venomous viper in Sweden. This prospective study investigated clinical signs, some biochemical parameters, treatment, and progress of disease after snakebite in 53 dogs. Effects of treatment with and without glucocorticoids were evaluated.

**Methods:**

All fifty-three dogs bitten by *Vipera berus *were examined the same day the dog was bitten and the next day. Two more examinations during 23 days post snake bite were included. Creatinine, creatine kinase (CK), alanine aminotransferase (ALT), glutamate dehydrogenase (GLDH), alkaline phosphatase (ALP) and bile acid results were followed through 3 to 4 samplings from 34 of the dogs.

**Results:**

All dogs had variable severity of local swelling in the bite area and 73 per cent had affected mental status. Initial cardiac auscultation examination was normal in all dogs, but six dogs had cardiac abnormalities at their second examination, including cardiac arrhythmias and cardiac murmurs. All dogs received fluid therapy, 36 dogs were given analgesics, 22 dogs were treated with glucocorticoids, and ten dogs were treated with antibiotics. Evidence of transient muscle damage (increased CK) was seen one day after the snake bite in 15 (54%) of 28 sampled dogs. Moderate changes in hepatic test results occurred in 1 dog and several dogs (22 of 34) had transient, minor increases in one or more hepatic test result. No dog died during the observation period as a consequence of the snake bite.

**Conclusions:**

Snake bite caused local swelling in all dogs and mental depression of short duration in most dogs. Some dogs had transient clinical signs that could be indicative of cardiac injury and some other had transient biochemical signs of liver injury. Treatment with glucocorticoids did not have any clear positive or negative effect on clinical signs and mortality.

## Background

Every year during the period April through September many dogs are bitten by *Vipera berus*, the only venomous snake in Sweden. *Vipera berus *belongs to the group of snakes called vipers. Vipers are widely distributed throughout Europe and Asia, from western Europe (Great Britain, Scandinavia, France) across central (Italy, Albania, Bulgaria and northern Greece) and eastern Europe to north of the Arctic Circle, and Russia to the Pacific ocean, Sakhalin, Island North Korea, northern Mongolia and northern China [1,2].

Despite the fact that *Vipera berus *envenomation is common in dogs and has a reported mortality of 3.5 - 4%, few investigations have addressed the clinical signs, biochemical findings and results of treatment [1,3,4]. The venom produced by *V. berus *is cytotoxic and stimulates production of cytokines, which can result in increased vascular permeability, vasodilatation and oedema. In serious cases the fluid loss from the capillary bed together with vasodilatation can cause severe hypovolaemia and distributive shock [5]. Therapeutic regimes used in Swedish veterinary medicine originate mainly from treatment recommendations in American textbooks concerning snakes related to the Swedish viper. The treatment often consists of a combination of intravenous fluids, glucocorticoids and antibiotics. The use of glucocorticoids, however, is controversial and its value has been questioned [4,6].

The main purposes of this prospective study were to better describe the clinical signs, to summarize evidence of organ damage based on physical exam and laboratory testing results and to evaluate treatment effectiveness especially with the question of use of glucocorticoids in treatment of snake bite with the Swedish viper.

## Methods

The study included dogs bitten by *Vipera berus *presented at Södra Djursjukhuset in Stockholm and at the University Animal Hospital at the Swedish University of Agricultural Sciences in Uppsala, Sweden, during the period April through August 2006. The inclusion criteria were a strong suspicion of viper bite at the time of presentation based on information from the dog owner (had seen the dog being bitten or seen a viper close to the dog) and/or clinical signs of a viper bite such as lethargy or swelling in the area of the suspected bite. Criteria for exclusion from the study were ongoing treatment with glucocorticoids for other reasons than the viper bite or a known history of liver disease.

The case history was obtained, including signalment (breed, age, sex, weight), time interval between the bite and presentation, and if any glucocorticoid treatment was given. Clinical data including mental status, body temperature, cardiovascular parameters (colour of mucous membranes, capillary refill time, heart rate/rhythm), the localisation of the bite and the degree of swelling were recorded. The degree of swelling was estimated by the examining veterinarian and the grade (none, minor, moderate or severe) was noted in a questioner prepared for this study. Mental status was based on if the dog was alert or slightly, moderately or severely depressed. Blood samples for the study were collected at presentation before therapy was initiated. The treatment at the two hospitals was adjusted to the patients' individual needs and was decided by the veterinarian on duty.

The dogs were examined, and if possible blood sampled, at four time points: on arrival (examination 1, 53 dogs), at 24 hours (examination 2, 52 dogs), and at follow-up on day 4-10 (examination 3, 46 dogs) and day 9-23 (examination 4, 33 dogs) after presentation. The time points for follow-up examinations and blood sampling were adjusted to suit the dog owners. Serum for the study was harvested and frozen (-20°C) until analysis at one time point one to five months after presentation.

The dogs were hospitalised for treatment and observation. Recording of ECG was not made in all dogs as a routine but was decided by the treating veterinarian. The duration of hospitalisation was decided based upon the clinical progression of the patient. The time for discharge from the clinic was decided by the treating veterinarian.

Approval of the study was obtained from the local Ethical Committee on Animal Experiments. Owner consent was given for all dogs studied.

### Serum biochemistry

In 34 of the total 53 dogs was it possible to collect serum at three or four examinations. In the remaining 19 dogs serum was only available from one or two time points, and these samples were not analysed and therefore excluded from the biochemical part of the study.

Serum concentrations of alanine aminotransferase (ALT), alkaline phosphatase (ALP), bile acids, glutamate dehydrogenase (GLDH), creatine kinase (CK) and creatinine were determined using an auto analyzer Konelab 30 (Thermo Clinical Labsystems Oy, Vantaa, Finland). Commercial reagents from Termo (Thermo) were used for all parameters, except for CK (DiaSys Diagnostics, Hotzheim, Germany), bile acids (Diazyme Laboratories, San Diego, US) and GLDH (Roche Diagnostics, Mannheim, Germany). There was no information if bile acids were pre or post prandial.

### Statistical analysis

One-way ANOVA was applied to evaluate differences in clinical and biochemical parameters between glucocorticoid treated group and the not glucocorticoid treated group (program JMP v5.0 [SAS, Cary, NC, US]). Sign rank test of median (Minitab 15, Minitab Ltd, Coventry, UK) was used to evaluate changes in biochemical parameters between the first sample within a few hours after the snake bite and the sample after 24 hours. No basal value was available because all four examinations were performed after the snakebite. Non-parametric methods were used because the distribution of the biochemical parameters was not parametric. Spearman rank correlation (Minitab 15) was used to evaluate correlation between swelling and mental status at the first examination and between swelling and CK-levels at the first two examinations.

The dogs were grouped according to whether they had received glucocorticoid therapy (GT) or not (NGT).

## Results

### Clinical findings

Of the 53 dogs, 32 were female (60%) and 21 were males (40%). The most common breeds were German shepherd (15%), Labrador retriever (9%), cross breed (8%), Flat coated retriever and Golden retriever (6% each). The ages of the dogs ranged from 3 months to 10 years, with a mean and median age of 4 years. The mean body weight was 25 kg (range 7 - 52 kg).

Four of the dogs were under treatment for other diseases at the time of the viper bite. One dog received synthetic thyroid hormone and trilostan for hypothyroidism and Cushing's disease, one dog received carprofen for back pain, one dog was treated with cefalexin and milbemycinoxim for demodicosis and atopic dermatitis and one dog was treated for external otitis with fusidindietanolamin, framycetin sulphate, nystatin and prednisolone. Further, one dog had a short period of lethargy and vomiting of unknown origin prior to the bite that resolved without treatment, and one dog had a one week old dog bite wound in the neck that was not treated.

Seventy-four per cent of the dogs were presented to the hospital within 3 hours of the snake bite and all dogs within 7 hours of the bite.

In 31 of the 53 dogs the owner observed the dog being bitten or a snake near the dog. In the remaining 22 dogs the signs and history were strongly indicative of a snake bite.

**Figure 1:**
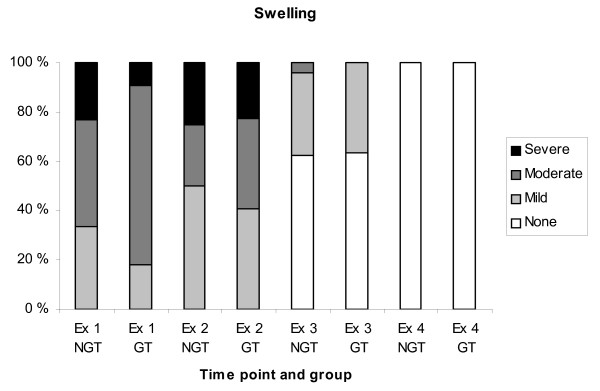
**Relative number of dogs with swelling around the area of the snakebite at the four examinations**. Ex = examination, NGT = Not glucocorticoid treated, GT = Glucocorticoid treated.

**Figure 2:**
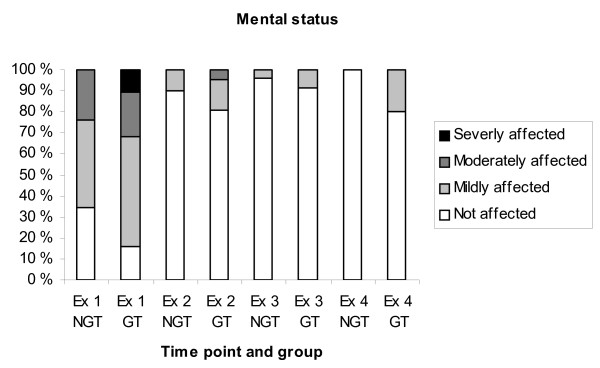
**Mental status at the four examinations**. Ex = examination, NGT = Not glucocorticoid treated, GT = Glucocorticoid treated.

All dogs had a varying degree of swelling in the area of the bite (Fig. 1). Most dogs were bitten in the head/nose (77%). Other locations were hind limb (13%), front limb (6%), neck and prepuce (2% each). On arrival at the hospital 73% of the dogs had an affected mental status (Fig. 2) and seven % of the dogs had an elevated body temperature (> 39.5°C). Cardiac variables were normal on auscultation in all dogs at examination 1. Spearman correlation between degree of swelling and degree of impairment of the mental status was low (r = 0.32).

All dogs except one were hospitalised for treatment and observation. All dogs received fluid therapy, consisting of crystalloid fluids (Ringer-Acetat, Fresenius Kabi, Homborg, Germany; Rehydrex with Glucose 2.5%, Fresenius Kabi, Homborg, Germany). The dosage ranged from 40-60 ml/kg/h to 40-60 ml/kg/day depending on the findings at the clinical examination on arrival. In some cases (28 dogs) the fluid therapy also included colloid fluids at dosages ranging from 5-20 ml/kg/10 min to 0.8 ml/kg/day (Haes-steril 60 mg/ml, Fresenius AG Bad Homburg, Germany; Voluven 60 mg/ml, Fresenius AG Bad Homburg, Germany).

Altogether 21 dogs (40%) were treated with glucocorticoids. Seven dogs (14%) were treated by the dog owner or referring veterinarian before arrival (Table 1). Fifteen dogs were treated on arrival at the hospital, two of these dogs had also been treated by the dog owner. Thirty-one dogs (60%) did not receive glucocorticoid treatment.

**Table 1:** Treatment with glucocorticoids pre or at admittance

	No dogs(%)		Number of treatments	Dosage
No treatment	31 (60%)	-	-	-

Pre admittance	5 (10%)	betamethasone p.o.^1^	Once	0,1-0,25 mg/kg
	1 (2%)	prednisolone inj^2^	Once	0,6 mg/kg
	1 (2%)	prednislone p.o^3^	Once	1 mg/kg

At admittance	11 (21%)	prednisolone inj^2^	Once	1-2 mg/kg
	2 (4%)	prednisolone inj^2^	2 -5 days	0,2-2 mg/kg
	3 (6%)	prednisolon p.o^3^	Once	1-1,25 mg/kg

Two of the dogs treated at admittance had earlier been treated by the dog owner.

^1 ^betamethasone (Betapred™ 0.5 mg, tablets, Swedish Orphan, Stockholm, Sweden)

^2 ^prednisolone acetat (Prednisolonacetat Vet. 10 mg/ml, injection, Intervet, Danderyd, Sweden)

^3 ^prednisolone (Prednisolon Pfizer, 2,5 mg, 5 mg, 10 mg, tablets, Pfizer AB, Sollentuna, Sweden)

Thirty-six of the 53 dogs (68%) were treated with analgesics including buprenorphine, methadone hydrochloride or transdermal fentanyl for 1-6 days. Ten of the 53 dogs (19%) were given antibiotics. Clinical signs related to infection of the snake bite were not seen in any dog. No dog received antiserum as part of the treatment. Sixty-nine % of the dogs was discharged after one day of observation and treatment (range 1-6 days).

Only 16% of the dogs still had an affected mental status and none had an elevated body temperature at examination 2. Swelling persisted around the area of the bite in all dogs and in some cases it was more pronounced than at the first examination (Fig. 2). Six dogs (11%) had cardiac abnormalities at examination 2, including cardiac arrhythmias, identified on auscultation, in five dogs (9%) and cardiac murmurs in two dogs (4%). The five dogs with arrhythmia were further evaluated with ECG and the arrhythmia was classified in two of the dogs as ventricular extra systoles in one dog and ventricular tachycardia in the other dog. These two dogs were treated with an anti-arrhythmic drug. Of the two dogs with cardiac murmur one was defined as physiological murmur at ultrasound, the other dog was not examined with ultrasound. Five of the six dogs with cardiac abnormalities had received glucocorticoids.

At examination 3 (day 4-10) some degree of swelling still remained in nine out of 35 dogs examined. At the fourth examination (day 9-23) there was no residual swelling in the region of the snake bite in any of the dogs (Fig. 1). In other respects the dogs were considered clinically normal at the third and fourth examinations except for one dog at the third examination, two dogs at the fourth examination and one dog at both the third and fourth examination, in which the mental status was still considered to be mildly affected.

No dogs died during the observation period as a consequence of the viper bite. One dog was euthanized during the observation period due to a suspected pulmonary neoplasia on x-ray but the diagnosis was not confirmed with autopsy.

### Differences in treatment groups

In most cases (83%) the degree of swelling was estimated before the start of treatment with glucocorticoids. At examination 1, the group of dogs treated with glucocorticoids showed a trend towards a greater degree of swelling compared with the group of NGT dogs, however, the difference was not significant. Neither was there any difference in the degree of impairment of the mental status between the treated and untreated group. Twenty-seven % of the dogs in the GT group displayed increased swelling at examination 2 compared with examination 1, whereas this proportion in the NGT group was 24 per cent. There was no significant difference between the two groups in other clinical parameters.

In the group of dogs treated with analgesics on admission, 20 per cent still had an affected mental status the day after arrival, in contrast to the group that did not receive analgesics, in which the mental status was unaffected in all dogs.

### Biochemical results

**Figure 3:**
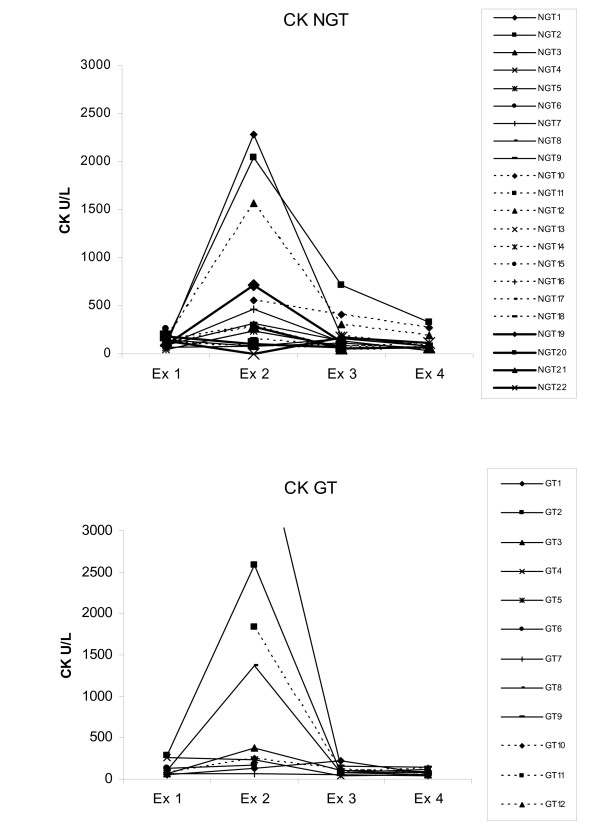
**CK values at the four examinations**. a) 22 dogs not treated with glucocorticoids (NGT = Not glucocorticoid treated) b) 12 dogs treated with glucocorticoids (GT = Glucocorticoid treated). Dog GT9 had CK concentrations of 3162 U/L at examination 1 and 4524 at examination 2. Reference value 240 U/L.

Evidence of muscle damage, seen as serum CK above reference values, was seen in 15 of the 28 sampled dogs (54%) at examination 2 (table 2). A pronounced increase (>1200 U/L) was seen in seven dogs (25%) (Fig. 3). In only three dogs did the CK values remain above the reference value at the third and fourth examination. There was no correlation (Spearmans correlation) between the degree of swelling and CK values at examination 1 and 2 (r = -0.14 and r = 0.07).

**Table 2:** Biochemical parameters at examinations 1 to 4 (Ex 1-Ex 4) for both groups.

	Untreated group				The glucocorticoid treated group			
	Ex 1	Ex 2	Ex 3	Ex 4	Ex 1	Ex 2	Ex 3	Ex 4
**CK**	N = 19	N = 18	N = 22	N = 15	N = 11	N = 10	N = 11	N = 12
Median	114	282	102	78	96	306	90	66
Range	(48-258)	(78-2280)	(48-714)	(36-330)	(54-3162)	(66-4524)	(42-222)	(42-222)
No. of dogs > reference value (240 U/L)	1	10	3	2	3	5	0	0
								
**GLDH**	N = 19	N = 18	N = 22	N = 15	N = 11	N = 10	N = 11	N = 12
Median	1980	2340	2160	2700	1680	1980	2040	1860
Range	(900-5520)	(1200-5880)	(660-41280)	(1260-4500)	(6600-12600)	(1380-6300)	(6900-8100)	(1500-8160)
No. of dogs > reference value (4500 U/L)	1	1	3	1	1	1	2	2
								
**ALT**	N = 19	N = 18	N = 22	N = 15	N = 11	N = 10	N = 11	N = 12
Median	30	36	30	42	48	51	48	39
Range	(6-66)	(12-84)	(6-276)	(12-84)	(30-108)	(30-78)	(30-120)	(24-348)
No. of dogs > reference value (72 μkat/L)	0	1	3	3	1	1	2	2
								
**ALP**	N = 17	N = 18	N = 22	N = 15	N = 11	N = 10	N = 11	N = 12
Median	78	93	93	96	78	210	210	153
Range	(24-378)	(54-366)	(24-1572)	(24-732)	(36-168)	(90-456)	(90-450)	(66-420)
No. of dogs > reference value (300 U/L)	1	2	4	3	0	2	2	1
								
**Bile acid**	N = 18	N = 18	N = 22	N = 15	N = 11	N = 10	N = 11	N = 12
Median	1.3	1.2	2.2	3.4	2.4	1.15	3.2	3.2
Range	(0.5-20.5)	(0.3-17.5)	(0.4-33.8)	(0.8-21.2)	(0.5-31.6)	(0.7-8)	(0.7-13.7)	(0.9-29.2)
No. of dogs > reference value (30 mmol/L)	0	0	1	0	1	0	0	0
								
**Creatinine**	N = 18	N = 18	N = 22	N = 15	N = 11	N = 10	N = 11	N = 12
Median	81	72	81	86	89	70.5	89	85.5
Range	(67-99)	(35-116)	(30-102)	(45-104)	(49-112)	(48-121)	(48-103)	(47-95)
No. of dogs > reference value (130 mmol/L)	0	0	0	0	0	0	0	0

The first four columns show the results from the group that was not treated with glucocorticoids (untreated). Ex = examination

**Figure 4:**
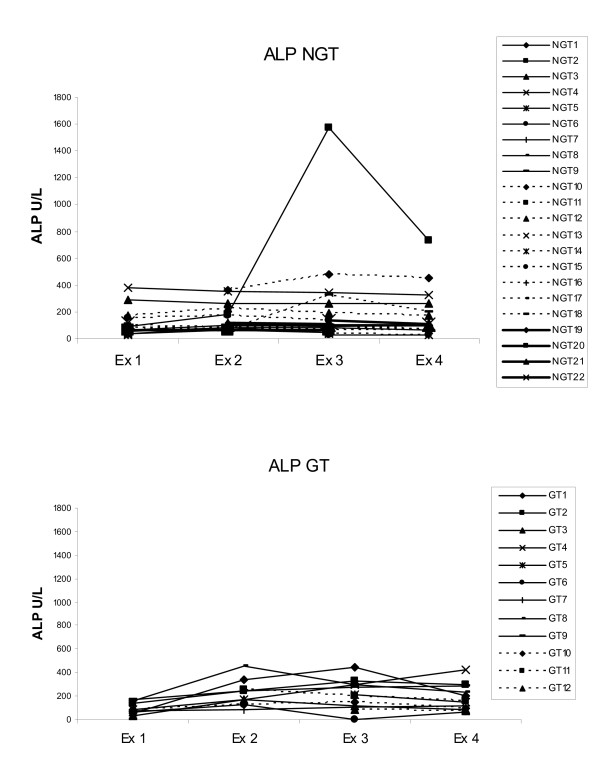
**ALP values at the four examinations**. 22 dogs not treated with glucocorticoids (NGT = Not glucocorticoid treated), 12 dogs treated with glucocorticoids (GT = Glucocorticoid treated). Dog NGT10 was only 3 months old, which may explain the continuous high ALP in that animal. Reference value 300 U/L. Ex = examination.

**Figure 5:**
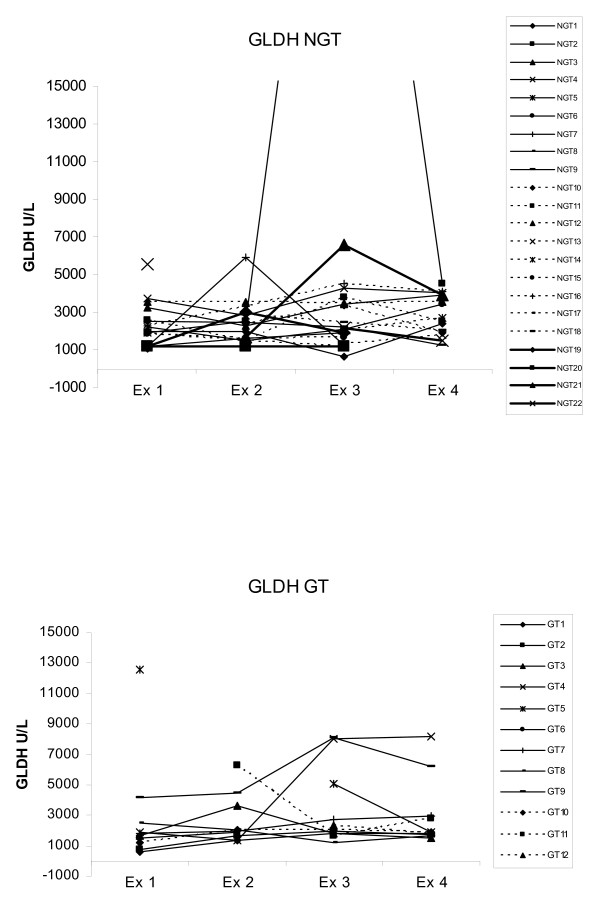
**GLDH values at the four examinations**. 22 dogs not treated with glucocorticoids (NGT = Not glucocorticoid treated), 12 dogs treated with glucocorticoids (GT = Glucocorticoid treated). Dog NGT2 had GLDH concentrations of 41280 U/L at examination 3. Reference value 4500 U/L.

There were biochemical signs of suspected transient hepatic injury in some dogs. Of the 34 sampled dogs 65% had serum concentration above reference values of at least one liver enzyme at one or more than one examination (Table 1, Fig. 4, Fig. 5).

Of these 34 dogs, 22 were not treated with glucocorticoids. In this NGT group, one dog had biochemical changes that strongly indicated liver damage, with a moderate increase in ALT, GLDH and ALP at examinations 3 and 4 (Fig. 4, Fig. 5). One other dog had results above reference values in GLDH and bile acids at examination 3 without any change in other parameters. Two further dogs had a slight increase in ALP at different time points without any increase in other liver parameters. There was also one dog with only a slight increase in GLDH at examination 1 and another dog with a high ALT value at examination 4.

Of the 34 dogs, 12 were treated with glucocorticoids and in this group a minor, but significant, increase in ALP was seen between examinations 1 and 2 (p < 0.05, sign. test) (Fig. 4). Four individuals had results slightly above reference values in GLDH (Fig. 5) and ALT at one or two examinations. In one dog GLDH and ALT were already elevated at presentation (examination 1). This dog had been treated with glucocorticoids by the owner two hours before arrival at the hospital. ALT was significantly higher (p < 0.05, ANOVA) in the glucocorticoid treated group than in the untreated group at the first sampling. No other differences in liver enzymes levels between the two groups were seen at any time point.

Serum creatinine was within reference values for all dogs at all examinations, except one dog that had slightly low creatinine values at examination 2 and 3. However, creatinine decreased from examination 1 to examination 2 in 82% of the sampled dogs (p < 0,05 Sign test).

## Discussion

At the first examination within a few hours after the snake bite all dogs had local swelling at the site of the bite and many had an affected mental status. After 24 hours the swelling persisted in all cases to some extent and had increased in a few cases. However by this point most dogs had shown clinical resolution of most other signs. However, some dogs showed transient signs that could be an indication of organ injury, affecting heart and liver. Cardiac arrhythmias developed in four dogs between examinations 1 and 2. No abnormalities were found at auscultation at examinations 3 and 4, and thus in our study there was no clinical evidence of permanent myocardial damage, although there was no biochemical tests run to evaluate this.

Clinical case observations had earlier suggested that dogs could develop hepatic disease after snake bites [1]. However, results of 4 different liver tests (ALT, ALP, GLDH and bile acids) analyzed at four time points after snake bite, spanning a period of 9-23 days, were inconsistently and often not greatly abnormal. Only one dog had biochemical evidence of hepatic injury, including both hepatocellular enzyme leakage and cholestasis. Many dogs had serum hepatic enzyme activities of one or more than one enzyme at one or more than one examination but these were only slightly above reference values. Mild inconstant increases in test values must be interpreted with caution. Reference values are based of the results from 95% of healthy dogs, so for each sampling 5% for each parameter are expected to be outside reference values. Thus 1/20 abnormal results may be high only by chance. Results however suggest occasional hepatic injury in some dogs and may be subclinical in more cases. The changes occurred both early (within 24 hours) and later at the third and fourth re-examination. In most cases the increase was temporary and only one or two parameters were affected. Hepatic injury may be from direct cytotoxic effects of snake venom or indirectly as a consequence of vascular damage and ischemia. In a study by Karlsson-Stiber C, Persson H, Heath A, et al mildly elevated values in ALT was seen in humans a few days following Vipera berus envenomation [7].

Glucocorticoids alone induce an increase in hepatic enzyme activity in dogs. In experimental studies it was found that dogs treated with 4 mg/kg dexamethasone showed a significant increase in ALP and ALT 48 hours after administration [8,9], whereas in a study by Peta et al. GLDH did not increase in most dogs receiving short-term prednisolone [10]. In the glucocorticoid treated group in our study there was a small, however significant, increase in ALP from sampling at occasion 1 to 2. This increase was probably induced by glucocorticoids, as it was not seen in the untreated group.

The elevated CK values at examination 2 were probably caused by local muscle damage at the site of the snake bite [11]. No evidence of renal failure was seen, however, the serum creatinine values decreased from examination 1 to examination 2 in 82% of the blood sampled dogs. The mild decrease might be explained by increased glomerular filtration caused by fluid therapy [12].

The biochemical changes in our study are consistent with those reported by other authors. Aroch and Harrus found generally mild biochemical changes in 109 dogs poisoned by *Vipera xanthina palestinae*, except for significant increases in CK and GLDH [3].

The sex distribution - 32 bitches and 21 males - is similar to that reported from other studies [1,3]. The most common breeds - German shepherd and Labrador retriever - reflect the dog population in Sweden [13]. A possible explanation for the age distribution (60% ≤ 4 years) may be that younger dogs are more curious and less careful than older dogs.

The most common location of the snakebite in the head and nose was similar to that previously reported in other studies concerning *Vipera palestinae *envenomation [3,4]. In the study by Segev et al., comprising 327 dogs bitten by *V. palestinae*, it was shown that the risk of mortality was increased when the bite was located in an extremity [6]. In our study there was no correlation between the site of the bite and the degree of affected mental status and no deaths occurred.

The use of glucocorticoids in viper bites is controversial and several authors have expressed doubts on the effect of this treatment [3,14,15]. The viper's venom contains enzymes that induce the production of endogenous cytokines and inflammatory mediators [1]. Glucocorticoids have an anti-inflammatory effect, inhibit capillary dilatation and prevent the release of vasoactive amines [16]. Experimental studies in dogs with septic shock have shown beneficial effects of treatment with glucocorticoids, but the results have been less convincing in human clinical trials, except in a subpopulation of human patients with relative adrenal insufficiency [17-20]. In a retrospective study including 327 dogs bitten by *V. palestinae *an increased mortality was found in dogs treated with glucocorticoids [6]. The authors pointed out that it could not be ruled out that it was the dogs with the most severe symptoms that were treated with glucocorticoids, and that this might have explained the increased mortality. In the present study there were no significant differences between the GT and NGT groups regarding clinical signs such as mental status, recovery period, or degree of local swelling. However, a slight trend towards a higher degree of swelling in the GT group than in the NGT group was noted. A possible reason for this could be that the severity of the clinical signs at presentation, including the degree of swelling, may have guided the veterinarians in their choice of treatment, so that dogs with a higher degree of swelling were more likely to receive glucocorticoids.

Of the dogs treated with analgesics on admission, 20 per cent still had an affected mental status on the day after arrival compared to those not treated with analgesics in all of which the mental status was considered to be unaffected. Again an explanation for this finding might be that the degree of affected mental status at presentation could have influenced the decision to administer analgesics. A sedative effect of the opioids provided can be another possible reason for the difference in mental status between the two groups. In the present study, no evaluation of pain was made.

The incidence of infection secondary to bites of *Vipera berus *in Swedish dogs is unknown and the benefit from antimicrobial therapy is debated. It has been reported that despite heavy oral and fang contamination of crotalid species with a wide variety of potentially pathogenic bacteria, crotalid envenomation in humans is associated with a low incidence of bacterial infection [21]. Our study also indicated that no clinical signs of infection were found in any dog despite that only 19% was treated with antibiotics.

No dog in the present study was treated with antiserum. Treatment with antiserum is the only way to inactivate the enzymes in the venom, by binding them to antibodies in the antiserum [19]. Antiserum should be considered in severely affected dogs in which supportive therapy is unsatisfactory [3,15,22,23]. In humans, the treatment has been shown to be effective for up to 18 hours after the bite (Internal document from the poison information centre in Sweden). Despite the fact that antiserum therapy was not used in the present study no mortality occurred. Further investigations of the effects of antiserum treatment in dogs bitten by *V. berus *are warranted.

The mortality reported in association with viper bites in dogs is similar in different studies. Kängström reported a mortality rate of 3.5 per cent in a retrospective study of 170 Swedish dogs bitten by *V. berus *[1], and Aroch and Harrus reported 3.7 per cent mortality in a retrospective study of 109 dogs poisoned by *V. xanthina palestinae *[3]. Segev et al. reported a mortality of 4 per cent in 327 dogs poisoned by *V. xanthina palestinae *[6]. Data from the largest animal insurance company in Sweden (Agria) showed a mortality of 2.9 per cent in 103 reported cases of viper envenomation in 2006 (Personal communication). This data does not include any information about treatment surrounding the snake bite. In our study there were no fatalities during the observation period as a consequence of the viper bite.

## Conclusions

In conclusion, the dogs in this study bitten by *Vipera berus *recovered from initial depression within 24 hours, whereas local swelling persisted for a longer period of time. Other clinical signs were mild and no deaths occurred during the follow-up period. Transient signs of liver injury and cardiac abnormalities were found in some dogs. It was difficult to draw any conclusions as to whether treatment with glucocorticoids had any significant effects on clinical signs and mortality.

## Competing interests

The authors declare that they have no competing interests.

## Authors' contributions

JBL participated in the design of the study, carried out the clinical study and drafted the manuscript. IL participated in the design of the study, carried out the biochemical part of the study, parts of the statistical analysis and drafted the manuscript. JF participated in the design of the study and drafted the manuscript. All authors read and approved the final manuscript.

## References

[B1] Kängström LE Huggormsbett hos hund och katt Svensk veterinärtidning 1989 41 (8-9) suppl. 19 38 46

[B2] McjDiarmid RW Campbell JA Touré T Snake species of the World: A Taxonomic and Geographic Reference Herpetologists' League 1999 1 397 403

[B3] Aroch I Harrus S Retrospective study of the epidemiological, clinical, haematological and biochemical findings in 109 dogs poisoned by vipera xanthina palestinae Veterinary Record 1999 144 19 532 5 10378282 10.1136/vr.144.19.532

[B4] Leisewitz AL Blaylock RS Kettner F The diagnosis and management of snakebite in dogs - a southern African perspective Journal of the South African Veterinary Association 2004 75 1 7 13 15214688 10.4102/jsava.v75i1.441

[B5] Karlsson-Stiber C Salmonson M Persson H A nationwide study of vipera berus bites during one year - epidemiology and morbidity of 231 cases Clinical Toxicology 2006 44 25 30 10.1080/15563650500394597 16496490

[B6] Segev G Shipov A Klement E Vipera palaestinae envenomation in 327 dogs: a retrospective cohort study and analysis of risk factors for mortality Toxicon 2004 43 6 691 699 10.1016/j.toxicon.2004.03.001 15109890

[B7] Karlsson-Stiber C Persson H Heath A First clinical experiences with specific sheep fab fragments in snake bite. Report of a multicentre study of Vipera berus envenoming Journal of Internal Medicine 1997 241 53 58 10.1046/j.1365-2796.1997.80896000.x 9042094

[B8] Syakalima M Takiguchi M Yasuda J Hashimoto A The age dependent levels of serum ALP isoenzymes and the diagnostic significance of corticosteroid-induced ALP during long-term glucocorticoid treatment The Journal of Veterinary Medical Science 1997 59 10 905 5 10.1292/jvms.59.905 9362039

[B9] DeNovo RC Prasse KW Comparison of serum biochemical and hepatic functional alterations in dogs treated with corticosteroids and hepatic duct ligation American Journal of Veterinary Research 1983 44 9 1703 1709 6137985

[B10] Peta HG Carr AP Myers SL Effect of serum storage, anti-inflammatory oral doses of prednisone, and spontaneous hyperadrenocorticism on serum glutamate dehydrogenase activity in dogs Veterinary clinical pathology 2007 36 1 25 29 10.1111/j.1939-165X.2007.tb00177.x 17311190

[B11] Lewis HB Rhodes DC Effects of I.M Injections of Serum Creatinine Phosphokinase (CPK) Values in Dogs Vet Clin Pathol 1978 7 11 12 10.1111/j.1939-165X.1978.tb00783.x 15334342

[B12] Sjaastad OV Hove K Sand O Physiology of domestic animals Scandinavian Veterinary press 2003 425 474

[B13] SKKs Registrerings-statistik http://www.hundsport.se/ring_rapport/skks_reg_statistik_mapp/reg_stat-topp20_2007.html

[B14] Aroch I Segev G Klement E Fatal vipera xanthina palestinae envenomation in 16 dogs Veterinary and Human Toxicology 2004 46 5 268 72 15487652

[B15] Garland T Recognition and treatment for snakebites Proceedings of the 18th annual American college of veterinary internal medicine. Seattle 2000 48 49

[B16] Maddison J Corticosteroids - friend or foe? Proceedings of the 34th World Small Animal Veterinary Congress WSAVA 2009 São Paulo, Brazil - 2009

[B17] White GL White GS Kosanke SD Therapeutic effects of prednisolone sodium succinate vs dexamethasone in dogs subjected to E. coli septic shock Journal of the American Animal Hospital Association 1982 18 639 648

[B18] Bennett IL Finland M Hamburger M The effectiveness of hydrocortisone in the management of severe infections Journal of the American Medical Association 1963 183 462 465

[B19] Lucas CE Ledgerwood AM The cardiopulmonary response to massive doses of steroids in patients with septic shock Archives of Surgery 1984 119 537 541 6712466 10.1001/archsurg.1984.01390170037008

[B20] Annane D Sébille V Charpentier C Effect of treatment with low doses of hydrocortisone and fludocortisone on mortality in patients with septic shock Journal of the American Medical Association 2002 288 7 862 71 10.1001/jama.288.7.862 12186604

[B21] Talan DA Citron DM Overturf GD Antibacterial activity of crotalid venoms against oral snake flora and other clinical bacteria The Journal of Infectious Diseases 1991 164 1 195 8 2056205 10.1093/infdis/164.1.195

[B22] Lobetti RG Joubert K Retrospective study of snake envenomation in 155 dogs from the Onderstepoort area of South Africa Journal of The South African Veterinary Association 2004 75 4 169 172 15830600 10.4102/jsava.v75i4.477

[B23] Hackett TB Wingfield WE Mazzaferro EM Benedetti JS Clinical findings associated with prairie rattlesnake bites in dogs: 100 cases Journal of American Veterinary Medical Association 2002 220 11 1675 80 10.2460/javma.2002.220.1675 12051509

